# A prognostic nomogram for event-free survival in patients with atrial fibrillation before cardiac resynchronization therapy

**DOI:** 10.1186/s12872-020-01502-4

**Published:** 2020-05-13

**Authors:** Minsi Cai, Wei Hua, Nixiao Zhang, Shengwen Yang, Yiran Hu, Min Gu, Hongxia Niu, Shu Zhang

**Affiliations:** Chinese Academy of Medical Sciences & Peking Union Medical College Fuwai Hospital, No. 167, Beilishi Rd, Xicheng District, Beijing, 100037 China

**Keywords:** Atrial fibrillation, Cardiac resynchronization therapy, Heart failure, Nomogram

## Abstract

**Background:**

Atrial fibrillation (AF), one of the most common comorbidities of heart failure (HF), is associated with worse long-term prognosis in HF patients receiving cardiac resynchronization therapy (CRT). However, there is still no convenient tool to identify CRT candidates with AF who are at high risk of mortality and hospitalization due to HF.

**Methods:**

We included 152 consecutive patients with AF for CRT in our hospital from January 2009 to July 2019. Multiple imputation was used for missing values. With imputed datasets, a multivariate Cox regression model was performed for variable selection using the backward stepwise method to predict all-cause mortality and HF readmissions. A nomogram and nomogram-based scoring system were constructed from the selected predictors. Then, internal validation and calibration were achieved by the bootstrap method, deriving the corrected concordance index and calibration curves. Sensitivity analysis was also performed to validate our selected predictors.

**Results:**

Five predictors were incorporated in the nomogram, including N-terminal pro brain natriuretic protein (NT-proBNP) > 1745 pg/mL, history of syncope, previous pulmonary hypertension, moderate or severe tricuspid regurgitation, thyroid-stimulating hormone (TSH) > 4 mIU/L. The concordance index (0.70, 95% CI 0.62–0.77), corrected concordance index (0.67, 95% CI 0.59–0.74) and calibration curve showed optimal discrimination and calibration of the established nomogram. A significant difference in overall event-free survival was recognized by the nomogram-derived scores for patients with high risk (> 50 points), intermediate risk (21–50 points) and low risk (0–20 points) before CRT.

**Conclusion:**

Our internally validated nomogram may be an applicable tool for the early risk stratification of CRT candidates with AF.

## Background

Cardiac resynchronization therapy (CRT) improves cardiac function and reduces morbidity and mortality in appropriately selected heart failure (HF) patients with sinus rhythm via biventricular pacing (BIVP) [1–3]. However, the efficacy of CRT in patients with atrial fibrillation (AF) still remains a knowledge gap, although the concurrence of AF and HF is common in clinical practice, ranging from < 5% in asymptomatic HF patients to nearly 50% in symptomatic HF patients [4]. Several studies have supported the benefits of CRT in AF patients [5–8], though compared with those in sinus rhythm, AF was also associated with a higher risk of mortality in CRT candidates [9–11]. Unfortunately, there are currently no predictive models proposed in clinical practice for AF patients undergoing CRT that can stratify patients by risk and evaluate their long-term prognosis. Therefore, we aimed to construct an easy-to-use nomogram to satisfy this urgent need. The nomogram incorporates baseline features before CRT as predictors and is effective for risk stratification among AF-HF patients.

## Methods

### Study design and population

We collected the medical records of all AF patients who underwent successful CRT or CRT-ICD (CRTD) for the first time at Fuwai Hospital between January 2009 and July 2019. AF subtypes were diagnosed according to criteria in the guidelines [12].

Patients who met the following criteria were included in the study: (1) without atrioventricular junction ablation (AVJA) before CRT/CRT-D or during follow-up; (2) without a history of successful radiofrequency ablation for atrial arrhythmia before CRT/CRT-D or during follow-up; and (3) with complete survival data. Finally, 152 patients were eligible for the data analysis (Online Fig. S1).

All patients received CRT implantation according to the guidelines [1, 13, 14]. Left ventricular (LV) pacing leads were preferably implanted in posterior-lateral, anterior-lateral or lateral veins through the coronary sinus. If implanting LV leads was not possible or failed, epicardial pacing would be the next choice. A successful CRT would entail the following: (1) all leads were fixed firmly at the target locations (right ventricular [RV] lead preferably at the apex; LV lead preferably in posterior/anterior lateral veins; atrial lead preferably at the right auricle but may not be implanted in permanent AF); (2) RV/LV lead with a pacing threshold ≤3.5 V/0.4 ms and atrial lead ≤1.5 V/0.4 ms; and (3) sensing and lead impedance in normal range defined by different pacemaker manufacturers. After implantation, parameter optimization on CRT was performed to achieve the shortest QRS duration for each individual patient. All patients were given individualized drug treatment at the discretion of doctors and clinical guidelines after discharge [1, 13, 14]. Follow-up was conventionally performed at 1, 3, 6 and 12 months after implantation during the first year and every 12 months subsequently. If there is a need or emergency, follow-up might be possible at any time. During each visit, the devices were interrogated: an experienced cardiologist and a technician of the pacemaker manufacturer checked basic parameters (sensing, lead impedance, threshold, etc.) and warnings on the programmer; pseudofusion would be identified by comparing QRS morphology and the location of the spike during each interrogation. Echocardiography and laboratory tests might be given, and VVI pacing mode, mode switch function or drug optimization would be initiated if necessary.

### Study endpoints

Patients with hospitalization due to heart failure (HFH) were defined as those with typical symptoms of heart failure (including paroxysmal nocturnal dyspnea, orthopnea, edema, faint or dizzy feelings, palpitation, etc.) who were presented to the hospital and stayed over 24 h, receiving at least 1 intravenous treatment (diuretics, inotropes, amiodarone, etc.) [15]. A composite endpoint was defined as the combination of all-cause mortality and HFH. Patients undergoing heart transplantation and left ventricular assist device implantation were defined as cardiovascular death.

### Data collection

The previous history and diagnosis, examination data and laboratory tests of all patients during hospitalization were obtained from the Electronic Medical Record System (EMRS) of Fuwai Hospital, including thyroid function test, liver and renal function test, electrolyte examination, NT-proBNP test, chest X-ray, echocardiography and electrocardiography.

Survival data, including hospitalization due to heart failure, causes of death and date for the endpoints, were retrieved not only from each follow-up and the EMRS, but also from contact with patients or their relatives via telephone or communication software.

All of the data were reviewed by two authors (MS, Cai and YR, Hu). Written informed consent was obtained before implantation and the study was approved by the ethics committee of Fuwai Hospital and adhered to the Declaration of Helsinki.

### Statistical analysis

Data analysis was performed in R version 3.6.0. Potential variables for model construction were based on a clinical priori and the findings of previous studies: age, sex, cardiac function class (NYHA class), AF type, complete left bundle branch block (CLBBB), complete right bundle branch block (CRBBB), intraventricular block (IVB), frequent premature ventricular contraction (fPVC, defined as an average of 10 or more PVCs per hour while monitored [16]), history of syncope, history of pulmonary hypertension, dilated cardiomyopathy (DCM), diabetes, coronary heart disease, pre-implantation echography parameters (including LVEF, mitral regurgitation (MR) and tricuspid regurgitation (TR)), creatinine, blood urea nitrogen (BUN),estimated glomerular filtration rate (eGFR), TSH, NT-proBNP, QRS duration (QRSd), cardiothoracic ratio and pulmonary congestion in X ray [9, 10, 17–22]. Continuous variables were described as the mean with standard deviation (SD) or median with interquartile range (IQR). Most of them were categorized based on conventional cutoff values in clinical practice (for example, 4 mIU/L for TSH) except for NT-proBNP, which was dichotomized based on maximally selected rank statistics [23]. Categorical variables were summarized as frequencies (%). Univariate Cox proportional hazards regression and K-M plot were used to find associations between variables and event-free survival. Variables included in multivariate Cox regression for further selection were those with a statistical significance of *P* < 0.2 or with clinical importance.

In terms of the Cox multivariate model, missing values were replaced by multiple imputation and then, predictor selection using backward stepwise regression with Akaike information criterion (AIC) on all imputed datasets was conducted. Final predictors would be variables remaining in nearly or over half of the models [24]. The proportional hypothesis was validated for the final model. Multicollinearity was evaluated by the variance inflation factor (VIF).

A nomogram based on the results of multivariate analysis was built with the rms package. The concordance index (C-index) was used to evaluate the discrimination ability of the nomogram, and calibration curves were used to assess the difference between the actual and predicted event-free survival rates using bootstrapping (500 resamplings) [25]. Internal validation was performed using bootstrapping (1000 resamplings) to avoid potential overfitting, and then a corrected C-index was given, which showed the future performance of our multivariate model for extrapolation. A risk scoring system based on the nomogram was constructed, and the total points of all patients were calculated. Patients were allocated to 7 groups according to their different scores, and subgroups with similar trends of event-free survival were merged.

Sensitivity analysis was performed in the following two ways. (1) An additional multivariate Cox model derived from complete cases (subjects without missing values for the selected predictors) in the same way as previously mentioned was used to validate the predictors in our nomogram. (2) TSH levels of patients with or without amiodarone intake were compared by the Mann-Whitney U test. A K-M plot was used to determine whether TSH still served as a predictor of survival in patients without amiodarone use. Finally, a multivariate predictive model was derived from complete cases without amiodarone intake. All of the above were used to rule out the influence of amiodarone on TSH levels. A comparison between the nomogram and a single predictor was performed to assess predictive accuracy.

## Results

### Baseline characteristics

One hundred and fifty-two patients were included in the analysis. The median age was 62 years (interquartile range (IQR), 54–69 years), and 41 patients were female (27%). Persistent or permanent atrial fibrillation was seen in 70 patients (46.1%), and paroxysmal atrial fibrillation was seen in 82 patients (53.9%). The median QRS duration before implantation was 160 ms (IQR,144–176.5 ms) with a median LVEF of 31.5% (IQR, 26–38%) and CLBBB in 53.3% of patients. Additionally, moderate or severe TR was present in 40 patients (26.3%), and pulmonary hypertension diagnosed before implantation was present in 29 patients (19.1%). A history of syncope was reported in 33 individuals (21.7%). With regard to laboratory tests, the median concentration of NT-proBNP was 1702 pg/mL (IQR, 1009.1–2612 pg/mL) and TSH was 2.32 mIU/L (IQR, 1.44–4.25 mIU/L). Other characteristics were also shown in Table 1.

**Table 1:** Baseline characteristics

Baseline	Value
**Demographic characteristics**	
Age (years, median [IQR])	62.00 [54.00, 69.00]
Female (%)	41 (27.0)
**Operation-related characteristics**	
CRT-D implantation (%)	83 (54.6)
Lateral/anterior-lateral/posterior-lateral LV lead (%)	119 (78.8)
Upgradation from PM/ICD (%)	17 (11.2)
**Comorbidities and disease history**	
Persistent/permanent AF (%)	70 (46.1)
DCM (%)	97 (63.8)
CLBBB (%)	81 (53.3)
IVB (%)	24 (15.8)
Coronary heart disease (%)	39 (25.7)
Myocardial infarction (%)	27 (17.8)
Hypertension (%)	52 (34.2)
Diabetes (%)	42 (27.6)
Hyperlipidemia (%)	50 (32.9)
NYHA III/IV (%)	110 (72.4)
AVB (%)	35 (23.0)
fPVC (%)	26 (17.1)
History of pulmonary hypertension (%)	29 (19.1)
History of PCI (%)	10 (6.6)
History of stroke (%)	20 (13.2)
History of CABG (%)	6 (3.9)
History of VT/VF (%)	60 (39.5)
History of syncope (%)	33 (21.7)
**Drug treatment**	
ACEI/ARB (%)	117 (77.0)
Beta receptor blockers (%)	128 (84.2)
Spironolactone (%)	124 (81.6)
Digitoxin (%)	80 (52.6)
Diuretics (%)	139 (91.4)
Statins (%)	70 (46.1)
Amiodarone (%)	47 (30.9)
Antiplatelets (%)	37 (24.3)
Warfarin (%)	32 (21.1)
NOAC (%)	30 (19.7)
**ECG**	
Preimplantation QRSd (ms, median [IQR])	160.00 [144.00, 176.50] (*n* = 144)
Postimplantation QRSd (ms, median [IQR])	144.00 [133.50, 160.00] (*n* = 143)
**Preimplantation chest X ray**	
Cardiothoracic ratio (median [IQR])	0.58 [0.55, 0.63]
Pulmonary congestion (%)	89 (58.6)
**Preimplantation echocardiography**	
LVEF (%, median [IQR])	31.50 [26.00, 38.00]
LVEDD (mm, median [IQR])	66.00 [60.00, 76.00]
Moderate/severe MR (%)	76 (50.0)
Moderate/severe TR (%)	40 (26.3)
**Preimplantation laboratory tests**	
NT-proBNP (pg/mL, median [IQR])	1702.00 [1009.10, 2612.00] (*n* = 149)
Total bilirubin (umol/L, median [IQR])	18.95 [13.77, 24.84] (*n* = 142)
Creatinine (umol/L, median [IQR])	93.30 [79.28, 112.20] (*n* = 151)
BUN (mmol/L, median [IQR])	7.58 [6.26, 9.57] (*n* = 151)
eGFR (mL/min/1.73m^2^ [IQR])	65.96 [51.60, 81.95] (*n* = 151)
TSH (mIU/L, median [IQR])	2.32 [1.44, 4.25] (*n* = 139)
**PM interrogation at last follow-up**	
BIVP (%, median [IQR])	98.65 [95.00, 99.00] (*n* = 132)
• Paroxysmal AF	99 [96.25, 99.00] (*n* = 70)
• Persistent/permanent AF	98 [92.25, 99.00] (*n* = 62)
• fPVC	99 [97.20,99.00] (*n* = 24)
**LVEF at 6–12 months after implantation (%, median [IQR])**	35 [30, 45] (*n* = 122)

*ACEI* angiotensin-converting enzyme inhibitor, *ARB* angiotensin II receptor blocker, *AVB* atrioventricular block, *BIVP* biventricular pacing, *CRT-D* cardiac resynchronization therapy with defibrillator, *CABG* coronary artery bypass grafting, *ICD* intracardiac defibrillator, *LVEDD* left ventricular end diastolic diameter, *NOAC* non-vitamin K antagonist oral anticoagulants, *PM* pacemaker, *PCI* percutaneous coronary intervention, *VT* ventricular tachycardia, *VF* ventricular fibrillation

### Treatment and survival

During hospitalization and after discharge, the patients were given individualized drug treatments, including ACEI/ARB (*n* = 117, 77%), beta-receptor blockers (*n* = 128, 84.2%), spironolactone (*n* = 124, 81.6%), digitoxin (*n* = 80, 50.2%), diuretics (*n* = 139, 91.4%), statins (*n* = 70, 46.1%), amiodarone (*n* = 47, 30.9%), antiplatelets (*n* = 37, 24.3%), warfarin (*n* = 32, 21.1%) and NOAC (*n* = 30, 19.7%).

**Fig. 1:**
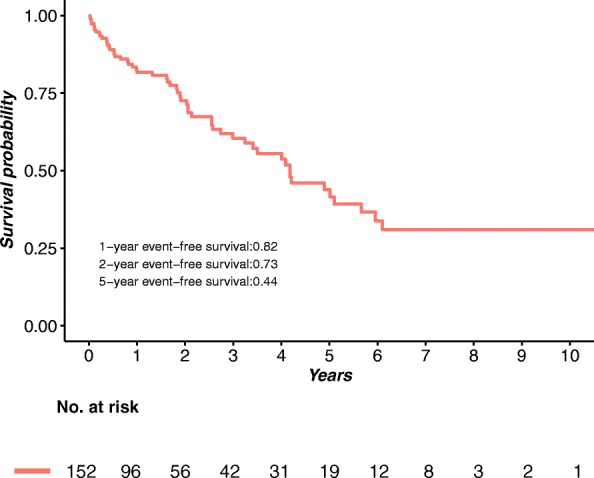
Kaplan-Meier event-free survival curve for all patients

The median follow-up was 578.5 days (range, 9–3888 days). During this period, 28 patients died (18.4%), 18 from cardiovascular causes (11.8%) and 10 from unexplained causes or other diseases (6.6%); 49 experienced HFH (32.2%), which resulted in 56 in total reaching the composite endpoint (36.8%). The event-free survival was 82, 73 and 44% at the 1-, 2- and 5-year follow ups, respectively (Fig. 1).

### Predictors and nomogram construction

**Fig. 2:**
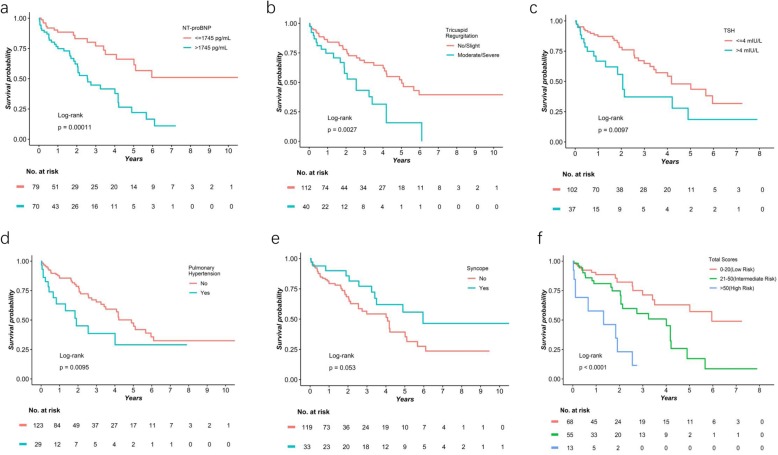
Kaplan-Meier event-free survival curves according to different predictors. **a** Event-free survival stratified by preimplantation N-terminal pro brain natriuretic protein concentration. **b** Event-free survival stratified by tricuspid regurgitation. **c** Event-free survival stratified by thyroid-stimulating hormone. **d** Event-free survival stratified by pulmonary hypertension. **e** Event-free survival stratified by history of syncope. **f** Event-free survival stratified by total scores from the established nomogram

**Fig. 3:**
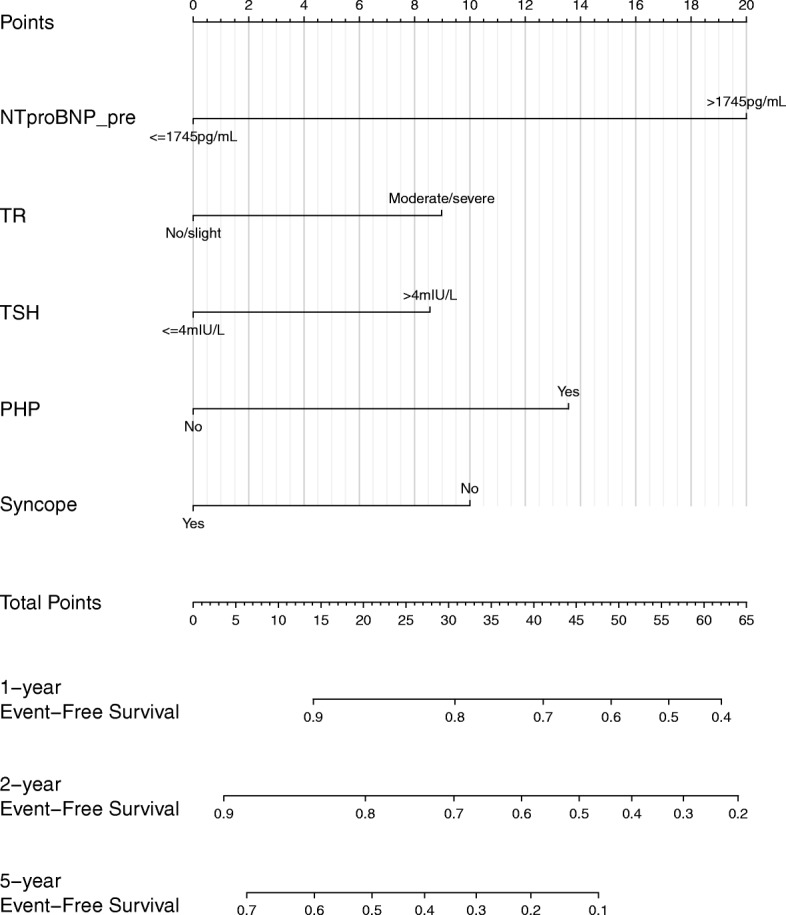
Nomogram for patients with CRT in AF. The nomogram is a commonly used prognostic prediction tool in the field of oncology. It can forecast the probability of a certain clinical event in the future. Doctors can easily use the nomogram with following steps: (1) affirm the value of each predictor for a patient on the variable axis; (2) draw a line upward to Points axis and the number at the intersection will be the points for each variable; (3) sum up all the points of each patient and locate the calculated total number on the Total Points axis; and (4) draw a line downward to different survival axes to determine final probabilities of a given clinical event. For example, in a patient with AF who had severe tricuspid regurgitation and an NT-proBNP concentration of 2000 pg/mL without other risk factors before CRT, then his total points would be approximately 29 points. Therefore, his event-free survival at 1, 2 and 5 years after CRT is estimated to be 81, 72 and 38% after CRT, respectively. NTproBNP_pre, N-terminal pro brain natriuretic protein concentration before CRT; PHP, history of pulmonary hypertension; TR, tricuspid regurgitation; TSH, thyroid-stimulating hormone

Five predictors were selected in a final model: preimplantation NT-proBNP > 1745 pg/mL (hazard ratio (HR) 2.47, 95% confidence interval (CI) 1.35–4.49, *P* = 0.0031), moderate/severe TR (HR 1.50, 95% CI 0.82–2.73, *P* = 0.1857), TSH > 4 mIU/L (HR 1.47, 95% CI 0.79–2.73, *P* = 0.2204), previous pulmonary hypertension (HR 1.84, 95% CI 0.98–3.48, *P* = 0.0595) and history of syncope (HR 0.64, 95% CI 0.32–1.27, *P* = 0.1993). The proportional hazard ratio hypothesis was ensured with no signs of multicollinearity judged by VIF < 2. Those predictors split the population into two groups with significant differences in event-free survival, except for a history of syncope with a marginal *P* value (Table 2, Fig. 2a-e). Then a nomogram was created according to the final multivariate Cox regression model (Fig. 3).

**Table 2:** Univariate and multivariate analyses

Variables	Univariate analysis		Multivariate analysis based on complete cases (***N*** = 136)		Multivariate analysis based on imputed datasets (***N*** = 152)		Points
	HR (95% CI)	***P***	HR (95% CI)	***P***	HR (95% CI)	***P***	
**Syncope**	0.52 (0.27–1.02)	*0.057*			0.64 (0.32–1.27)	*0.199*	10 (*No syncope*)
**Pulmonary Hypertension**	2.19 (1.19–4.04)	*0.011*			1.84 (0.98–3.48)	*0.060*	14
**Moderate or severe TR**	2.32 (1.32–4.06)	*0.003*	2.02 (1.07–3.80)	*0.029*	1.50 (0.82–2.73)	*0.186*	9
**TSH > 4 mIU/L**	2.14 (1.19–3.87)	*0.012*	1.89 (1.03–3.46)	*0.040*	1.47 (0.79–2.73)	*0.220*	9
**NT-proBNP > 1745 pg/mL**	2.93 (1.66–5.17)	*< 0.001*	2.32 (1.23–4.38)	*0.009*	2.47 (1.35–4.49)	*0.003*	20
**CLBBB**	0.66 (0.39–1.12)	*0.120*	0.56 (0.31–1.01)	*0.054*			
**LVEF < =35%**	1.64 (0.87–3.11)	*0.130*					
**IVB**	1.51 (0.81–2.81)	*0.196*					
**Pulmonary congestion**	2.14 (1.19–3.87)	*0.012*					
**Cardiothoracic ratio**							
**< =0.5**	–	*–*					
**0.51–0.60**	1.72 (0.52–5.68)	*0.371*					
**> 0.61**	3.15 (0.95–10.46)	*0.062*					

*CLBBB* complete left ventricular bundle branch block, *IVB* intraventricular block, *LVEF* left ventricular ejection fraction, *NT-proBNP* N-terminal pro brain natriuretic protein, *TR* tricuspid regurgitation, *TSH* thyroid-stimulating hormone

### Performance of the nomogram

**Fig. 4:**
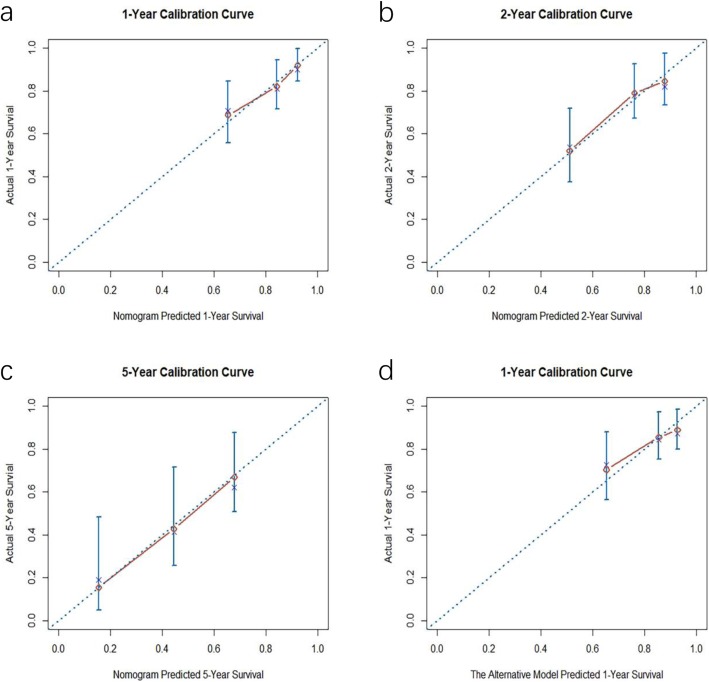
Calibration curves at different time points. Red lines represent the correlation between actual values and predictive values. Diagonal dashed lines represent the most perfect prediction. The cross signatures represent corrected predictive values versus actual values. **a** One-year calibration curve. **b** Two-year calibration curve. **c** Five-year calibration curve. **d** One-year calibration curve for the alternative model based on complete cases

The C-index of the established nomogram was 0.70 with a 95% CI of 0.62–0.77. Furthermore, calibration curves showed a moderate correlation between the predicted event-free survival and actual survival rates at the 1-, 2- and 5-year follow-ups (Fig. 4a-c). Regarding internal validation, the corrected C-index was 0.67 with a 95% CI of 0.59–0.74.

### Risk stratification of patients

The total points of each patient were calculated from the nomogram-derived scoring system (Table 2). Subjects with different points were assigned to 7 subgroups (points: 0–10, 11–20, 21–30, 31–40, 41–50, 51–60, > 60), and those with similar event-free survival curves were merged (Online Fig. S2). Therefore, this population was divided into 3 groups with different risks of the composite endpoints, of which patients with > 50 points were defined as high risk, 21–50 points as intermediate risk and 0–20 points as low risk (Fig. 2f).

### Alternative model based on complete cases

A new model based on complete cases was also created (Table 2) and CLBBB (HR 0.56, 95% CI 0.31–1.01), NT-proBNP > 1745 pg/mL (HR 2.32, 95% CI 1.23–4.38), moderate/severe TR (HR 2.02, 95% CI 1.07–3.80) and TSH > 4 mIU/L (HR 1.89, 95% CI 1.03–3.46) were included. The C-index was 0.70 with a 95% CI of 0.61–0.78, and the corrected C-index was 0.67 with a 95% CI of 0.60–0.75 after internal validation. Calibration curves at 1,2 and 5 years after implantation demonstrated inferior agreement between the prediction and actual survival compared with the nomogram prediction (Fig. 4d; Online Fig. S3).

### The influence of amiodarone on TSH level

Considering the possible influences of amiodarone on TSH levels, the TSH concentration of patients with and without amiodarone use were compared, but no statistical significance was found (2.69 [1.57, 3.80] mIU/L vs 2.27 [1.42, 4.28] mIU/L, *P* = 0.853). After patients receiving amiodarone were excluded, TSH > 4 mIU/L was still a predictor for event-free survival (Online Fig. S4). Finally, the significant multivariate model based on complete cases without using amiodarone also included TSH > 4 mIU/L as a predictor, with a *P* value equal to 0.0952.

### Nomogram versus a single independent predictor

As shown in Table 2, NT-proBNP > 1745 pg/mL was an independent risk factor for survival. Therefore, the predictive performance of the established nomogram and NT-proBNP was compared. The C-index for event-free survival prediction was 0.62 (95% CI, 0.55–0.69) by NT-proBNP only, significantly lower than the indices of the nomogram (0.70, 95% CI 0.62–0.77, *P* = 0.0036).

## Discussion

The long-term beneficial effects of CRT on overall survival and hospital admissions due to heart failure have been illustrated by previous randomized control trials (RCTs) [2, 3]. However, patients included in RCTs were all in sinus rhythm before CRT implantation, which ignored the fact that AF coincided with HF in 5% of asymptomatic patients and in nearly 50% of symptomatic patients [26]. Furthermore, AF worsened the survival probability of patients with HF and was indicated to be an independent risk factor for poor prognosis after CRT [10, 27, 28]. Recent guidelines have given Class IA recommendations to subjects who need ventricular pacing and suffer from a high degree of AVB; AF patients have also been included [1]. However, there is no convenient tool to identify CRT candidates with AF who are at high risk of mortality and heart failure readmissions after implantation. The nomogram we proposed, to the best of our knowledge, is the first easy-to-use predictive model to satisfy this urgent need. Patients with estimations of more than 50 points in our model are predisposed to poor prognosis, so more rational decisions such as AVJA or intensive drug treatment and frequent follow-up should be considered for them.

Our nomogram included five predictors from conventional examinations and tests before CRT. NT-proBNP > 1745 pg/mL was the only independent risk factor in the multivariate model, where a history of pulmonary hypertension had a marginal significance. Moderate/severe TR, TSH > 4 mIU/L and history of syncope were not independent predictors. The nomogram established based on this multivariate Cox model showed optimal discrimination (C-index 0.70, 95% CI 0.62–0.77) and calibration. The corrected C-index from internal validation also demonstrated fair discrimination (0.67, 95% CI 0.59–0.74).

Additionally, sensitivity analysis based on complete cases confirmed the predictive value of moderate/severe TR, TSH > 4 mIU/L and NT-proBNP > 1745 pg/mL, which was in accordance with previous findings [17, 19, 21, 22, 29]. Notably, increased TSH levels are indicative of overt and subclinical hypothyroidism, which are related to a higher risk of impaired endothelial function and cardiac systolic and diastolic dysfunction posing negative impacts on general prognosis [30]. Experimental evidence also implied that hypothyroidism could increase AF susceptibility in rats due to a longer atrial effective refractory period and left atrial fibrosis associated with thyroid dysfunction [31]. Although history of pulmonary hypertension was not included in the new model, its independent risk on the composite endpoint was reported in patients with CRT [18, 20]. Thus, it was reasonable as a predictor in the nomogram. Similarly for history of syncope, patients with syncope from arrhythmias or low perfusion were believed to benefit from CRT, explaining its eligibility in the nomogram [1]. Moreover, the new model had inferior agreement between the actual and predicted survival rates compared with the model from imputed datasets. When compared with the univariate model including NT-proBNP, the model from imputed datasets showed significantly better predictive accuracy. Therefore, our nomogram was developed on the model from imputed datasets, and the nomogram-derived scoring system successfully performed risk stratification for patients with distinct long-term event-free survival.

Interestingly, the type of AF was not associated with the prediction of composite endpoint of HF patients, possibly due to benefits from optimal drug treatments after discharge and a high percentage of biventricular pacing (BIVP, median > = 98%), which was consistent with previous conclusions [15, 32]. Even though much data have recommended AVJA for patients undergoing CRT in persistent or permanent AF to ensure better BIVP and prognosis, few studies have explored the effects of paroxysmal AF on long-term prognosis [11]. One study indicated that paroxysmal AF increased the risk of mortality by 32% after adjustment for age, sex, BIVP% and shock treatment of CRT-D compared with 51 and 28% in persistent and permanent AF, respectively [32]. Therefore, AF types may possibly vary in episode duration by definition, but they are supposed to be equally assessed in patients with CRT because clinical diagnosis of AF subtypes was reported to lack accuracy in reflecting AF temporal persistence [33]. Our nomogram implied that various AF subtypes of patients may be regarded evenly in the prediction.

In addition, some previously accepted outcome predictors in CRT recipients were not valid in our nomogram, including CLBBB, QRS duration, ischemic or nonischemic cardiomyopathy, renal dysfunction and MR [1, 10]. This discrepancy may be explained by the following reasons. (1) Predictors such as QRS duration and CLBBB are potentially related to the prognosis of patients in sinus rhythm rather than AF according to current guidelines [1, 14]. Because all patients of our study were in AF, it seems plausible to obtain different results. Even for CRT candidates in sinus rhythm, they were also reported as lacking predictive accuracy for mortality and HFH [34]. (2) The conception of ischemic or nonischemic cardiomyopathy has been historically used interchangeably with a spectrum of diseases, so we did not consider them as potential predictors in univariate analysis but instead used similar and more specific diagnoses (e.g., coronary heart disease and DCM) [14]. (3) Our retrospective study with a limited population may account for this phenomenon, and the results should be interpreted with caution, although a predictive model derived and validated in large prospective cohorts did not include most of these variables either [35].

Our study has several limitations. First, this is a single-center retrospective study with a small sample size. Nonetheless, our center is the largest tertiary hospital of cardiovascular diseases in China, and patients coming from other provinces receive CRT implantation here, which to some extent, augments the extrapolation of our nomogram. Second, the therapeutic effects were not considered in our analysis because regular investigation of daily drug treatment for patients is not practical in our single center. Additionally, during the long study period from 2009 to 2019, indications for CRT, implantation techniques and examination technology have been updated, so the baseline of our patients may not be totally standardized. A limited inclusion timeframe would be favored in future studies on this topic. Finally, our nomogram has not been validated by external cohorts. Therefore, more studies need to be performed to test the validity of our established nomogram.

## Conclusion

In summary, we constructed an easy-to-use and internally validated nomogram containing 5 baseline predictors before CRT. This nomogram allows physicians to evaluate and predict outcomes of CRT recipients in AF and identify subjects with high risk to achieve better prognosis. This nomogram should be applied and validated in external cohorts with larger sample sizes.

## Supplementary information

**Additional file 1: Figure S1.** Flow chart of the study. **Figure S2.** Original Kaplan-Meier event-free survival curves for different risk scores derived from the nomogram. **Figure S3.** Calibration curves at different time points for the alternative model based on complete cases. Red lines stand for correlation between actual values and predictive values. Diagonal dashed lines represent the most perfect prediction. The cross signature stands for corrected predictive values versus actual values. **a**: 2-year calibration curve for the alternative model based on complete cases. **b**: 5-year calibration curve for the alternative model based on complete cases. **Figure S4.** Kaplan-Meier event-free survival curves stratified by TSH level in patients without amiodarone intake

## Data Availability

The datasets used and/or analysed during the current study are available from the corresponding author on reasonable request.

## Abbreviations

ACEI/ARB: Angiotensin-converting enzyme inhibitor/angiotensin II receptor blocker
AF: Atrial fibrillation
AVB: Atrioventricular block
BIVP: Biventricular pacing
BUN: Blood uretic nitrogen
CRT/CRT-D: Cardiac resynchronization therapy/cardiac resynchronization therapy with defibrillator
CABG: Coronary artery bypass grafting
CLBBB: Complete left bundle branch block
DCM: Dilated cardiomyopathy
eGFR: estimated glomerular filtration rate
EF: Ejection fraction
fPVC: frequent premature ventricular contraction
HF/HFH: Heart failure/hospitalization due to heart failure
ICD: Intracardiac defibrillator
IVB: Intraventricular block
LV: Left ventricle
LVEDD: Left ventricular end diastolic diameter
MR: Mitral regurgitation
NYHA: New York heart association
NOAC: Non-vitamin K antagonist oral anticoagulants
NT-proBNP: N-terminal pro brain natriuretic protein
PM: Pacemaker
PCI: Percutaneous coronary intervention
QRSd: QRS duration
TR: Tricuspid regurgitation
TSH: Thyroid stimulating hormone
VT: Ventricular tachycardia
VF: Ventricular fibrillation
