# Supplementary material for: A prognostic nomogram for event-free survival in patients with atrial fibrillation before cardiac resynchronization therapy
Source: BMC Cardiovasc Disord. 2020 May 13;20:221. doi: 10.1186/s12872-020-01502-4 (PMC7222436; doi:10.1186/s12872-020-01502-4)

Online resource: A nomogram-based score for prognosis in patients with atrial fibrillation before cardiac resynchronization therapy

Minsi Cai, MD1, Wei Hua, MD, FHRS1, Nixiao Zhang, MD1, Shengwen Yang, MD1, Yiran Hu, MD1, Min Gu, MD1, Hongxia Niu, MD1, Shu Zhang, MD, FHRS1

1 State Key Laboratory of Cardiovascular Disease, Fuwai Hospital, National Center for Cardiovascular Diseases, Chinese Academy of Medical Sciences and Peking Union Medical College, Beijing, 100037, People's Republic of China

Corresponding author:

Hua Wei, MD, FHRS

E-mail address: drhuaweifw@sina.com

ORCID: http://orcid.org/0000-0003-4014-989X

Fig. S1 Flow chart of the study

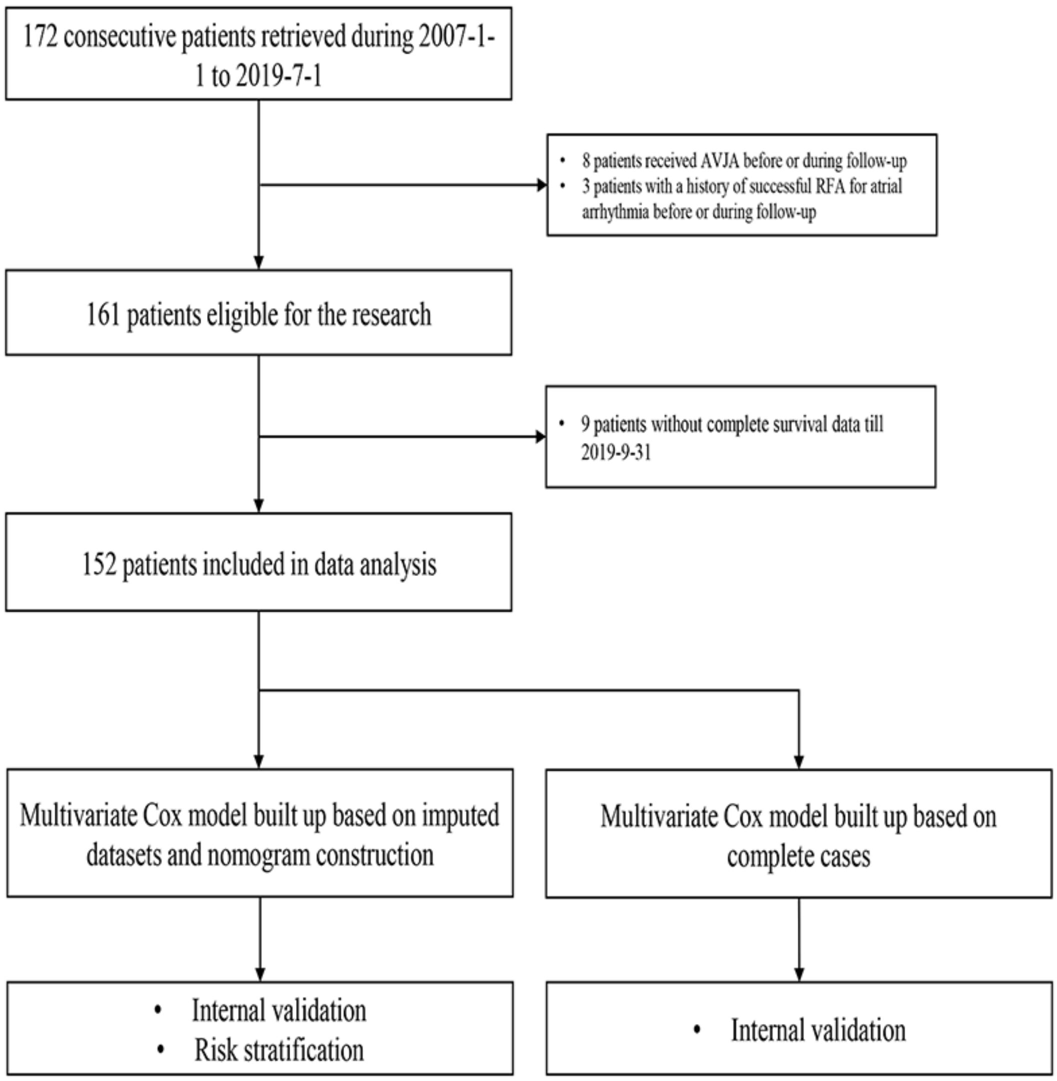

Fig. S2 Original Kaplan-Meier event-free survival curves for different risk scores derived from the nomogram

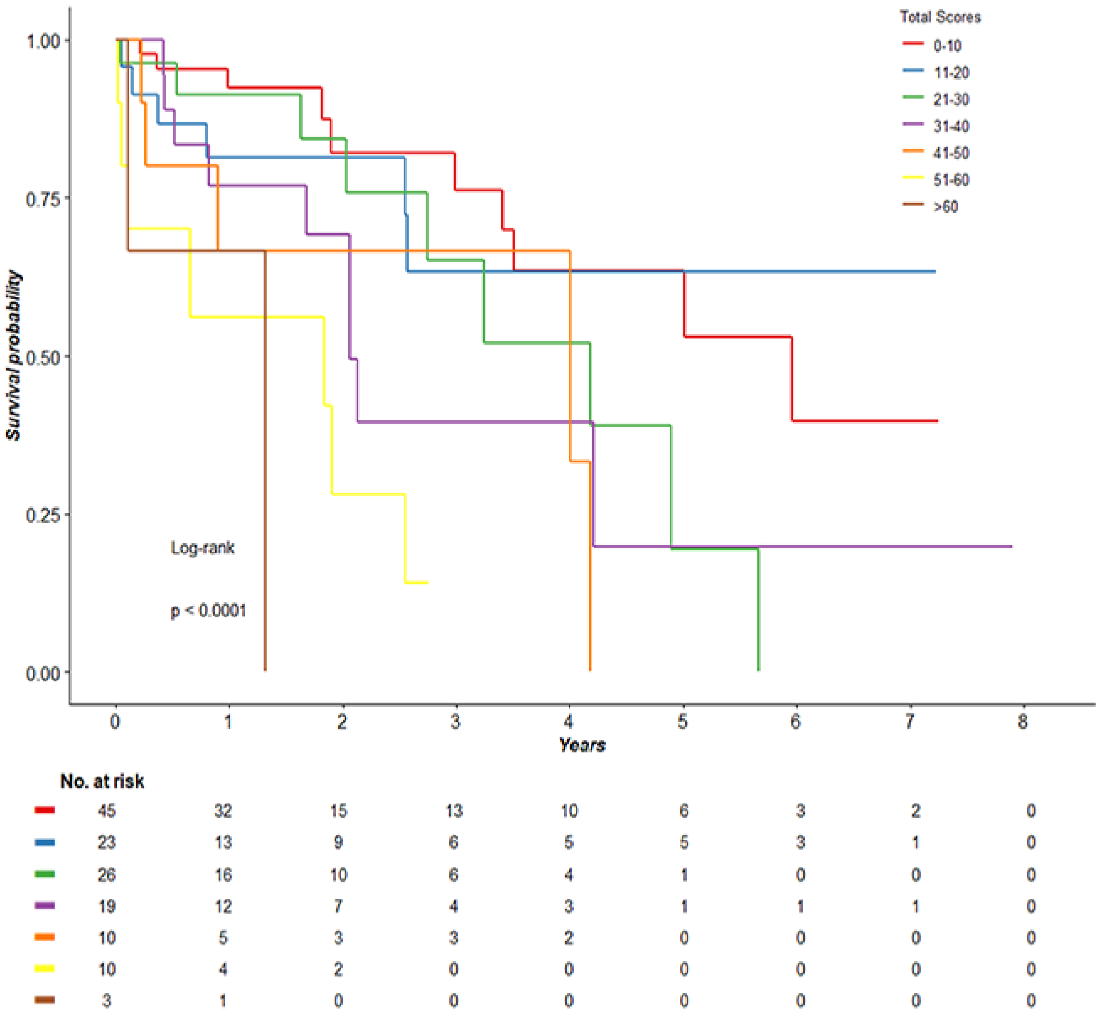

Fig. S3 Calibration curves at different time points for the alternative model based on complete cases. Red lines stand for correlation between actual values and predictive values. Diagonal dashed lines represent the most perfect prediction. The cross signature stands for corrected predictive values versus actual values. **a:** 2-year calibration curve for the alternative model based on complete cases. **b:** 5-year calibration curve for the alternative model based on complete cases

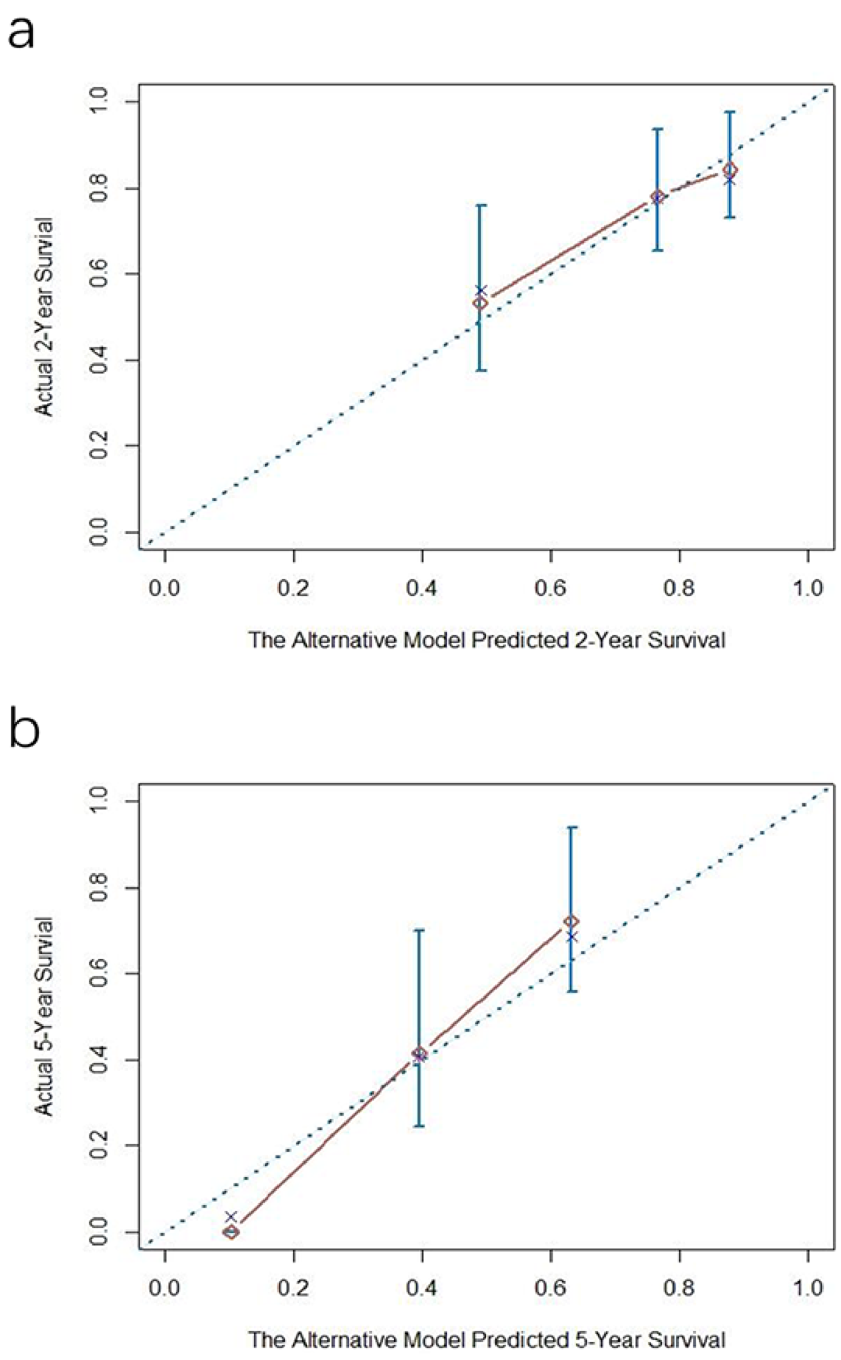

Fig. S4 Kaplan-Meier event-free survival curves stratified by TSH level in patients without amiodarone intake

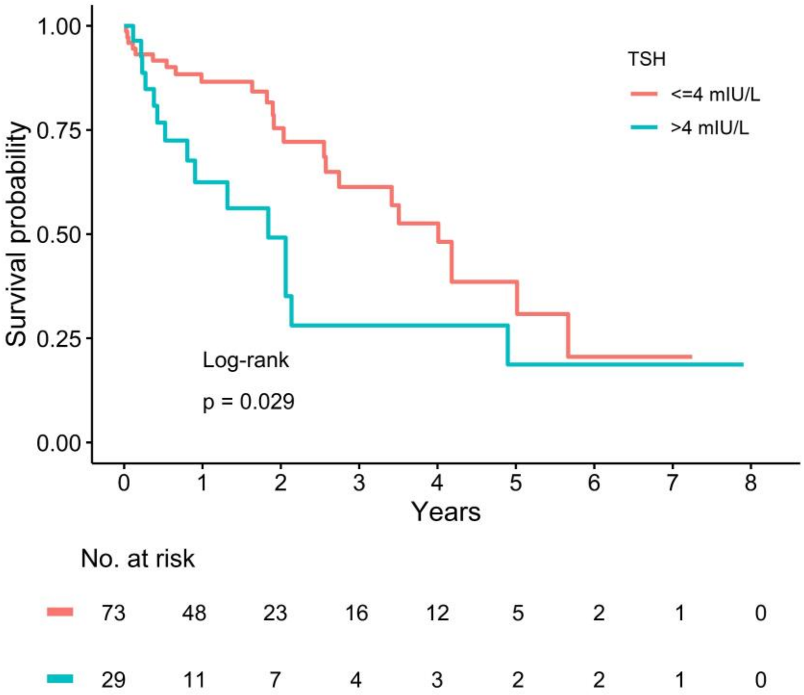

Supplement: Supplementary file 1 — Additional file 1: Figure S1. Flow chart of the study. Figure S2. Original Kaplan-Meier event-free survival curves for different risk scores derived from the nomogram. Figure S3. Calibration curves at different time points for the alternative model based on complete cases. Red lines stand for correlation between actual values and predictive values. Diagonal dashed lines represent the most perfect prediction. The cross signature stands for corrected predictive values versus actual values. a: 2-year calibration curve for the alternative model based on complete cases. b: 5-year calibration curve for the alternative model based on complete cases. Figure S4. Kaplan-Meier event-free survival curves stratified by TSH level in patients without amiodarone intake [file 12872_2020_1502_MOESM1_ESM.pdf]
